# Membrane Tubulation with a Biomembrane Force Probe

**DOI:** 10.3390/membranes13120910

**Published:** 2023-12-18

**Authors:** Lancelot Pincet, Frédéric Pincet

**Affiliations:** 1 Institut des Sciences Moléculaires d’Orsay, Université Paris-Saclay, CNRS, F-91405 Orsay, France; lancelot.pincet@universite-paris-saclay.fr; 2 Laboratoire de Physique de l’École Normale Supérieure, ENS, Université PSL, CNRS, Sorbonne Université, Université Paris Cité, F-75005 Paris, France

**Keywords:** tubulation, BFP, micromanipulations, GUV, force

## Abstract

Tubulation is a common cellular process involving the formation of membrane tubes ranging from 50 nm to 1 µm in diameter. These tubes facilitate intercompartmental connections, material transport within cells and content exchange between cells. The high curvature of these tubes makes them specific targets for proteins that sense local geometry. In vitro, similar tubes have been created by pulling on the membranes of giant unilamellar vesicles. Optical tweezers and micromanipulation are typically used in these experiments, involving the manipulation of a GUV with a micropipette and a streptavidin-coated bead trapped in optical tweezers. The interaction forms streptavidin/biotin bonds, leading to tube formation. Here, we propose a cost-effective alternative using only micromanipulation techniques, replacing optical tweezers with a Biomembrane Force Probe (BFP). The BFP, employing a biotinylated erythrocyte as a nanospring, allows for the controlled measurement of forces ranging from 1 pN to 1 nN. The BFP has been widely used to study molecular interactions in cellular processes, extending beyond its original purpose. We outline the experimental setup, tube formation and characterization of tube dimensions and energetics, and discuss the advantages and limitations of this approach in studying membrane tubulation.

## 1. Introduction

Tubulation, which is the formation of a membrane tube of 50 nm to 1 µm in diameter, is a common process in cells [1,2]. It is notably used to form intercompartmental connections and to transport material from one place to another. Tubes are also found between cells and are used for content exchange [3,4,5,6]. Because of their high curvature, tubes can also be used as specific targets for proteins that sense local geometry and use the curved membrane as a substrate where they perform their action. Hence, tubulation is a process that is absolutely necessary in trafficking and information transfer within the cell.

Similar tubes have been formed in vitro by pulling on the membrane of a giant unilamellar vesicle (GUV) [7,8,9,10,11,12,13]. They have been extensively used as platforms to study the action of membrane-modeling proteins. In parallel, the energetics of tube pulling have been theoretically described [14,15,16]. Usually, the experiment involves using a mix of micromanipulation and optical [8,9,10,11,13] or magnetic [12] tweezers. A GUV with a small fraction of biotinylated lipids is manipulated with a micropipette. A streptavidin-coated bead is trapped in optical tweezers. Bringing the bead and GUV in contact for a few seconds is sufficient to ensure the formation of several streptavidin/biotin bonds. Upon separating the GUV from the bead, the streptavidin/biotin bonds remain formed, forcing a contact point between the GUV membrane and the bead. To maintain this contact point while separating the GUV, a tube is formed. The position of the bead in the optical or magnetic tweezers indicates the force exerted by the tube on the bead. This technique, with both micromanipulation and tweezers, is rather expensive and requires multiple kinds of expertise. Here, we propose an alternate, cheaper solution in which only micromanipulation techniques are involved. The concept underlying the approach is to use a Biomembrane Force Probe (BFP) instead of optical tweezers. The BFP was designed in the 90’s by Evan Evans to probe the energy landscape of single molecular bonds [17,18,19,20]. It relies on a biological nanospring: a micromanipulated biotinylated erythrocyte. A streptavidin bead is bound to the erythrocyte through streptavidin/biotin bonds and used to probe forces in the local environment. The stiffness of the spring can be controlled over three orders of magnitude, from 10 to 10,000 pN/µm, by adjusting the aspiration in the micropipette that holds the erythrocyte. Since the minimum erythrocyte deformation that can be measured is ~10 nm and the erythrocyte can be deformed beyond ~1 µm, forces in the range of 0.1 pN to 10 nN can be detected in theory. Practically, the reasonable range of accessible forces is 1 pN to 1 nN which is the standard range of forces involved in physiological processes. The BFP provided unprecedented insights in molecular interactions involved in many processes, such as cellular adhesion or inflammation. It has been extended far beyond its original purpose and is now used to study the kinetics of ligand–receptor binding [21,22], cell membrane organization [23,24,25], cell–cell interactions [26,27], protein network crosstalk [28,29] and cell networks [30,31], or in mechano-biology in general [32]. It can also be combined with fluorescence to locally observe changes in molecular or membrane features [33]. Here, we show how the BFP can easily be used for controlled membrane tubulation. We first present how to prepare the experiment and form tubes. Then, we show how to characterize the tube dimensions and the energetics involved. Finally, we discuss the advantages and limitations of this approach.

## 2. Materials and Methods

### 2.1. Erythrocyte Biotinylation

To build the probe, erythrocytes needed to be biotinylated. This was achieved by a series of washing and incubation steps. All washing was carried out with centrifugation at 3–4000× *g* for 2 min and resuspension of the pellet. An amount of 2 to 3 µL of blood was washed in 1 mL PBS, pH 7.4, ~300 mOsm, to isolate the erythrocytes, and subsequently washed with carbonate–bicarbonate buffer, pH 8.5, ~180 mOsm. Alkaline pH and lower osmolarity that stretches the membrane accelerates the reaction between NHS and amine groups. The reactant solution was prepared during the erythrocyte washing by dissolving 2 mg NHS-PEG-biotin (e.g., EZ-Link™ NHS-PEG4-Biotin, product # A39259 from Thermo Fisher, Illkirch Graffenstaden, France) in 1 mL carbonate–bicarbonate buffer, pH 8.5, ~180 mOsm. After the last wash, the erythrocyte pellet was resuspended with the NHS-PEG-biotin solution and incubated under gentle aspiration at room temperature for 1 h.

After incubation, the erythrocyte solution was washed three times in Tris buffer, pH 7.5, ~300 mOsm. Tris buffer blocks the unreacted remaining NHS groups, and the physiological osmolarity ensures that the erythrocytes recover their native discocyte shape. The erythrocytes were kept under gentle agitation in Tris for ~30 min. After a last of round of three washes in PBS, pH 7.4, ~300 mOsm, the biotinylated erythrocytes were ready to use and could be stored in PBS at 4 °C for one week.

### 2.2. Streptavidin Beads

Streptavidin silica beads are commercial. In the results presented here, we used silica beads from Bangs Laboratories, Inc. (product #CS01002, Fishers, IN, USA). An alternate and cheaper solution is to directly coat the glass beads with streptavidin. Below is a standard protocol.

Glass beads of the right diameter, 2–5 µm, are commercially available and cheap. The procedure is a 4-step process: cleaning, silanization and biotinylation of the bead followed by the coating of a single layer of streptavidin.

The cleaning of the glass bead is achieved by boiling it in H_2_O_2_ (30%) with 50 drops of ammonia solution (final pH ~10.9) for 5 min. The bead solution is then washed 3 times in pure water with 0.01% sodium azide to avoid bacterial growth. If needed, glass beads can be stored at 4 °C.

For silanization of the glass beads, a maximum density of hydroxyl groups must be reached on the surface. Hence, if the beads were not cleaned just before silanization, it is advised to boil them for 5 min, which increases the density of hydroxyl groups. Then, the beads are washed three times in methanol. The beads are resuspended in a solution made of methanol:acetic acid:water:N-(2-aminoethyl)-3-aminopropyltrimethoxysilane (92:1:4:3 vol/vol). N-(2-aminoethyl)-3-aminopropyltrimethoxysilane can be bought from Sigma-Aldrich (Product #8191720100, Saint Quentin Fallavier, France). The bead solution is then incubated for 1 h under gentle agitation at room temperature. After incubation, the beads are washed three times in methanol and, after the last wash, resuspended with two drops of methanol. After drying these two drops, the dried beads are desiccated overnight. The following day, the beads are resuspended in methanol and progressively resuspended in PBS, pH 7.4, ~300 mOsm, by washing them with PBS:methanol solution with gradually less methanol (100%, 80%, 60%, 30%, 10%, no methanol). The silanized beads can be stored at 4 °C.

Biotinylation is achieved by following a protocol similar to the biotinylation of the erythrocytes described above. Streptavidin coating is applied by incubation of the beads in a solution of streptavidin at 0.5% (*w*/*w*) in water for 30 min. The beads are subsequently washed three times and resuspended in water with sodium azide.

### 2.3. Giant Unilamellar Vesicle Preparation

GUVs are prepared following the standard electroformation protocol [34]. In brief, 1 µL drops of lipid chloroform solution at 1 mM are deposited on two indium–tin oxide (ITO)-coated glass plates at 30 °C. The ITO plates are placed facing each other, separated by a washer made, for instance, of PDMS. The assembled plates are placed in a desiccator for 1 h. A solution of sucrose solution at known osmolarity, usually ~300 mM to be close to physiological osmolarities, is then injected between the two ITO plates. Two electrodes are connected to the two ITO plates, allowing for the application of increasing 10 Hz AC tension in 6 min steps: 100 mV, 200 mV, 300 mV, 500 mV, 700 mV, 900 mV, 1.2 V. The AC tension remains at 1.2 V for 1 h. A final 1.4 V step at 4 Hz is applied for 30 min and the plates can be stored in the fridge for a few hours before GUV harvesting. The osmolarity of all solutions used during the experimental procedures must be up to 20% higher than the osmolarity of the sucrose solution to avoid breaking of the GUVs.

### 2.4. Micropipette Formation

Two micropipettes are needed for tube formation with the BFP. The first one is 2–5 µm in diameter to hold the GUV and the other one is 1 to 2 µm in diameter and holds the erythrocytes. Micropipettes are made in a standard two step procedure. They are initially pulled from 1 mm glass capillaries in a pipette puller and subsequently forged in a microforge to shape their tip. In a Sutter Instruments P-2000 micropipette puller, the standard settings are:

For the large micropipette:

- HEAT = 350, FIL = 4, VEL = 55, DEL = 255, PUL = 255.

For the small micropipette, a two-cycle setting is preferable:

- HEAT = 350, FIL = 5, VEL = 40, DEL = 50, PUL = 40
- HEAT = 350, FIL = 5, VEL = 40, DEL = “blank”, PUL = 255

### 2.5. Lipids

All lipids were bought from Avanti Polar lipids. In the experiments presented here, the lipid composition was:

POPC:DOPS:DSPE-PEG2000-Biot:DOPE- Atto488: 84:10:5:1.

POPC (product #850457), DOPS (product #840035), DSPE-PEG2000-Biot (880129) and DOPE, respectively, stand for 1-palmitoyl-2-oleoyl-glycero-3-phosphocholine; 1,2-dioleoyl-sn-glycero-3-phospho-L-serine; 1,2-distearoyl-sn-glycero-3-phosphoethanolamine-N-[biotinyl(polyethylene glycol)-2000]; and 1,2-dioleoyl-sn-glycero-3-phosphoethanolamine. DOPE-Atto488 was bought from Atto-Tec. It is critical to have the PEG linker in the biotinylated lipid to ensure strong binding with streptavidin. When there is no PEG linker, the biotin moiety is too close to the membrane, preventing complete insertion in the streptavidin pocket.

## 3. Results

### 3.1. How to Form a Membrane Tube with the Biomembrane Force Probe

In the setup, two micropipettes are facing each other under an inverted microscope. One micropipette, with a 1–2 µm diameter, is for the BFP, and the other one, with a 2–4 µm diameter, is holding a GUV. These micropipettes are positioned on two micromanipulators that allow accurate positioning of the GUV and the BFP and filled with a solution of the same osmolarity as the buffer in which the experiment is performed. Using the same buffer in the experiment chamber and in the micropipette is usually a convenient solution. The other extremities of the micropipettes are connected to tubing that are attached to water reservoirs. These reservoirs are placed on vertical micro-translations that can be manual or motorized. Moving the reservoirs changes the hydrostatic pressure at the tip of the micropipettes and therefore the aspiration on the GUV or the BFP. This aspiration allows for precise control of the surface tension of the GUV and the erythrocyte, providing that the membrane does attach to the micropipette glass wall. This is a classical issue in micromanipulation experiments: correct transduction of the aspiration is absolutely required to quantitatively analyze the measurements. A standard protocol to avoid the binding of membranes on the glass wall (and the coverslip) is to preincubate the chamber and the micropipette with a 10% bovine serum albumin (BSA) or 1% β-casein solution for ~1 h. The easiest way to empirically test the membrane is not binding to glass wall to ensure that the end of the elongation inside the micropipette remains a half-sphere when decreasing the aspiration to zero. If there is adhesion, the half-sphere flattens upon retraction of the elongation.

Once the micropipettes are passivated with BSA or β-casein and the chamber is filled with the experiment buffer, biotinylated erythrocytes, streptavidin-coated beads and GUVs can be added to the solution. Biotinylating erythrocytes is a standard procedure for which several protocols have previously been published. We describe an efficient biotinylation procedure in the Materials and Methods. Streptavidin beads are commercially available (e.g., from Bangs Laboratories) and should be a few µm in diameter. If needed, we also present a protocol to coat glass beads with streptavidin. Making GUVs is also standard. Electroformation is a technique that provides many clean GUVs purely made of lipids. If proteins need to be added to the GUVs during their formation, electroformation does not always keep their activity or embed them in the membrane at high density. Alternate protocols have previously been published to prepare such proteo-GUVs. In any case, biotin moieties must be present on the GUV, through a biotinylated lipid or protein. GUVs are made in a buffer denser than that used in the experiment, ensuring that the GUVs, like the erythrocytes and the beads, settle down at the bottom of the chamber.

As we shall see below, it is critical to accurately determine the “zero” pressure that corresponds to the height of each reservoir at which there is neither aspiration nor suction in the corresponding micropipette. To find this height, $h_{0}$, it is convenient to gently aspirate a contrasted particle from the solution inside the micropipette and slowly move the reservoir up and down until the particle is immobilized (refer to Video S1). $h_{0}$ will slightly change if evaporation occurs during the course of the experiment and, therefore, may need to be regularly measured. When the reservoir is displaced to a new height, h, below $h_{0}$, the aspiration inside the micropipette, ∆P, is:

$$∆P=\left(h_{0}-h\right)\rho g$$

where ρ is the density of the solution in the reservoir and tube (pure water) and g is the gravitational acceleration. Hence, 1 mm displacement corresponds to approximately 10 Pa.

**Figure 1:**
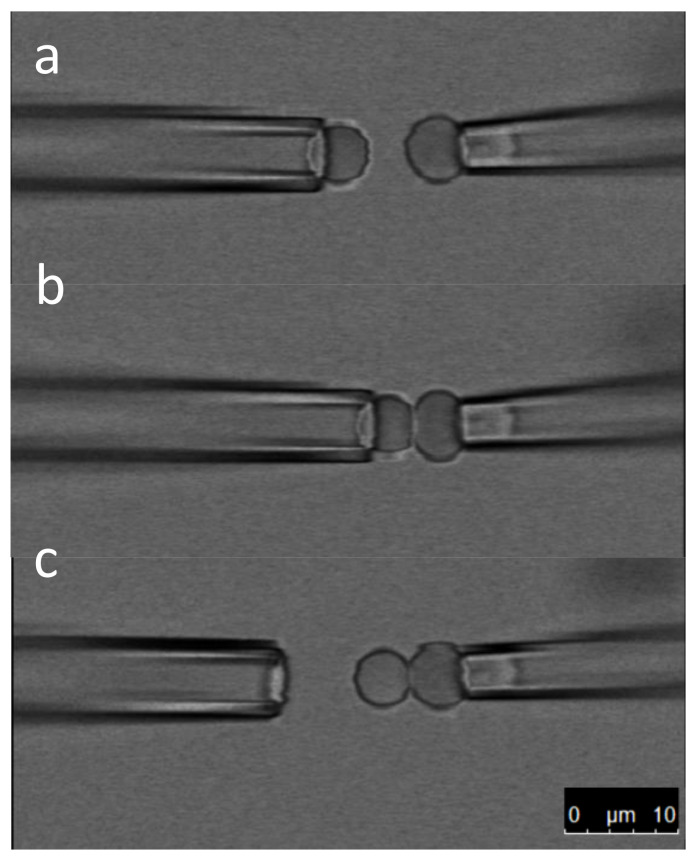
BFP formation. The three snapshots are extracted from Video S1. In panel (**a**), a streptavidin-coated bead is securely held by the left micropipette, while a biotinylated erythrocyte is held by the right micropipette. In panel (**b**), the bead is brought into contact with the erythrocyte, facilitating the formation of streptavidin/biotin bonds. Finally, in panel (**c**), the bead is released from the left pipette, resulting in the assembly of the probe comprising the erythrocyte and the bead. This assembled probe is now ready for use in force measurements.

To make the BFP, one of the micropipettes holds a bead and the other an erythrocyte with low aspiration (10–50 Pa). The two micropipettes are then aligned and brought together so that the bead touches the erythrocyte membrane (Figure 1, Video S2). Waiting for a few seconds allows for the formation of several streptavidin/biotin bonds. The bead can then be gently released from the micropipette. The BFP is ready to use. It is important that the bead and erythrocyte are aligned with the axis of the micropipette to make sure the force is correctly transduced. This axial symmetry must be preserved as much as possible during the course of an experiment.

It is critical that the protrusion of the erythrocyte is larger than the radius of the holding micropipette to ensure accurate determination of the surface tension that is used to obtain the BFP nanospring stiffness (see Equation (6) below). Once this criterion is checked, the stiffness is obtained by measuring the radius of the erythrocyte, $R_{e}$, the radius of the micropipette, $R_{p}$, and the radius of the bead/erythrocyte contact, $R_{c}$, through [35]:

(1a) $$k_{e}=\frac{\pi R_{p}∆P}{\left(1-\frac{R_{p}}{R_{e}}\right)ln\left(\frac{4R_{e}^{2}}{R_{p}R_{c}}\right)-\left(1-\frac{R_{p}}{4R_{e}}-\frac{3R_{p}^{2}}{8R_{e}^{2}}+\frac{R_{c}^{2}}{R_{e}^{2}}\right)}$$

which is often approximated by [17,20]:

(1b) $$k_{e}=\frac{\pi R_{p}∆P}{\left(1-\frac{R_{p}}{R_{e}}\right)ln\left(\frac{4R_{e}^{2}}{R_{p}R_{c}}\right)}$$

The force magnitude is contingent on the selected expression, whether Equation (1a) or Equation (1b), while the relative variations remain invariant regardless of the chosen formulation. The force exhibits a linear trend, with an accuracy of 5% for deformations up to around 300 nm and within 10% for deformations up to approximately 500 nm. However, the primary emphasis generally lies on studying the relative force values, and working at larger erythrocyte deformations minimally influences the drawn conclusions.

The inherent discocyte shape of erythrocytes at rest is attributed to the cortical cytoskeleton, which induces anisotropic surface tension. To validate the accuracy of the BFP stiffness expressions presented in Equation (1), it is imperative that the surface tension remains isotropic. This isotropy is confirmed by the spherical morphology of the erythrocyte section extending beyond the micropipette. Achieving this requires adequate aspiration in the micropipette to overcome the surface energy imposed by the cytoskeleton. Additionally, it is notable that over their 120-day lifespan in the blood circulation, erythrocytes exhibit a tendency to progressively decrease in size. Consequently, the dimensions of the spherical cap outside the micropipette are contingent upon the age of the erythrocyte and necessitate meticulous measurement.

Once the BFP has been assembled, a GUV is grabbed by the second micropipette and brought within the vicinity of the bead; the axes of the two micropipettes must coincide as observed in the microscope. In reality, the two axes are at an angle because the two pipettes are slightly facing down to reach the bottom of the chamber. This small tilt affects the quantitative measurement with a cosine which remains negligible in most cases. The GUV is then moved towards the bead. Once the erythrocyte is compressed, indicating a repulsive force, the GUV can be retracted away from the bead, possibly after a waiting time. If streptavidin/biotin bonds were created during the GUV/bead contact, a tube will spontaneously form. Monitoring the position of the bead during the whole cycle provides a direct measurement of the force generated by the tube. Once a tube is formed, it is possible to repeat approach/separation cycles and possibly change the surface tension of the GUV by varying the aspiration in the GUV micropipette.

### 3.2. How to Measure the Tube Diameter

**Figure 2:**
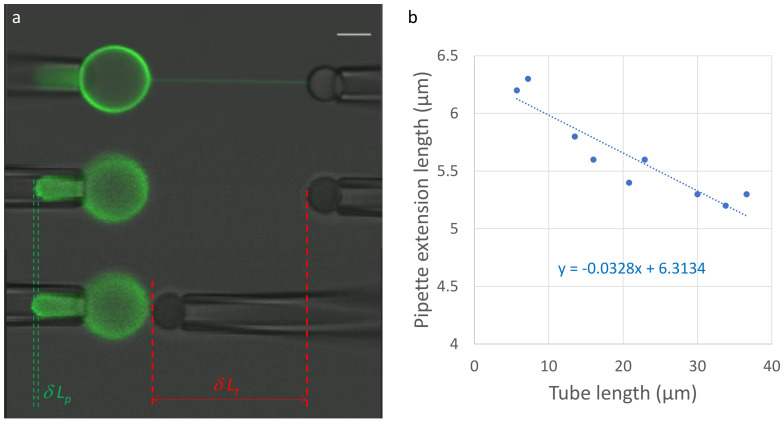
Tube diameter from tube length variations. After tube formation, the tube diameter is gauged by altering the tube length through a controlled approach and separation of the two micropipettes (panel (**a**)). In the upper image, the microscope is focused on the tube, while in the lower two images, it is focused on the end of the vesicle extension within the micropipette. Given that the vesicle surface area and the volume of the vesicle lumen remain constant, changes in tube length ($\delta L_{t}$) and vesicle extension in the micropipette ($\delta L_{p}$) directly quantify the tube diameter using Equation (4). In panel (**b**), data points are presented for two approaches and one separation, where the slope is proportionate to the tube diameter. In this specific instance, the tube diameter measures 82 nm, with an accuracy of approximately 10%.

During a tubulation experiment, the total volume of the lumen (inside) and the total membrane surface area remain constant. Hence, varying the distance between the two micropipettes results in an opposite variation in the tube length, $\delta L_{t}$, and the membrane protrusion inside the GUV micropipette, $\delta L_{p}$ (Figure 2a, Video S3). Assuming a spherical shape of the GUV, with a diameter of $d_{v}$, the total volume and membrane surface area can be, respectively, well approximated as:

(2) $$\left\{\begin{array}{c} V=\frac{\pi }{12}d_{p}^{3}+\frac{\pi }{4}d_{p}^{2}L_{p}+\frac{\pi }{6}d_{v}^{3}+\frac{\pi }{4}d_{t}^{2}L_{t}\\ A=\pi d_{p}L_{p}+\frac{\pi d_{p}^{2}}{2}+\pi d_{v}^{2}-\frac{\pi d_{p}^{2}}{4}+\pi d_{t}L_{t} \end{array}\right.$$

Upon varying the distance between the micropipettes, these equations lead to the following variations in the total volume and membrane surface area:

(3a) $$\left\{\begin{array}{c} \Delta V=\frac{\pi }{4}d_{p}^{2}\delta L_{p}+\frac{\pi }{2}d_{v}^{2}\delta d_{v}+\frac{\pi }{4}d_{t}^{2}{\delta L}_{t}=0\\ \Delta A=\pi d_{p}{\delta L}_{p}+2\pi d_{v}\delta d_{v}+\pi d_{t}\delta L_{t}=0 \end{array}\right.$$

which can be written as follows:

(3b) $$-2d_{v}^{2}\delta d_{v}=d_{p}^{2}\delta L_{p}+d_{t}^{2}{\delta L}_{t}={d_{v}d}_{p}{\delta L}_{p}+d_{v}d_{t}\delta L_{t}$$

Assuming that $d_{t}\ll d_{v}$ (which is always true), Equation (3b) leads to:

(4) $$d_{t}=-\frac{\delta L_{p}}{\delta L_{t}}d_{p}\left(1-\frac{d_{p}}{d_{v}}\right)$$

Making several approach/separation cycles provides an accurate measurement of the tube diameter (Figure 2b, Video S3). The absence of hysteresis between the approach and separation also shows that there is no measurable adhesion of the GUV on the micropipette. Adhesion would alter the shape of the membrane in the protrusion which would make the actual calculation of the variations in the total volume and surface area much more difficult. In the example of Figure 2 and Video S3, two approaches and one separation perfectly overlap (Figure 2b), showing the absence of hysteresis. The pipette diameter is 2.7 µm. Since the slope is −0.033, the tube diameter is ~82 nm. The accuracy of this value can be estimated from the fit of the approach/separation cycles (Figure 2b) and is usually close to 10%.

**Figure 3:**
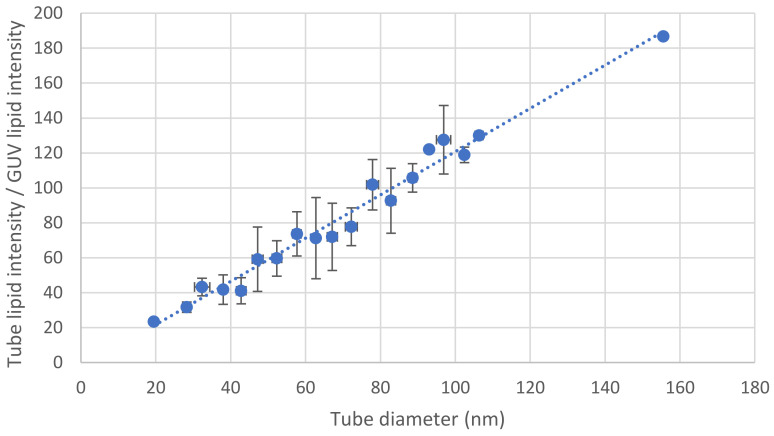
Tube diameter from fluorescence intensity. For a specific fluorescent lipid, the ratio, *r*, between the intensity of the tube and the intensity at the equator of the GUV is directly proportional to the tube diameter. The method outlined in Figure 2 is employed to measure the diameter for various tubes, enabling the determination of the proportionality coefficient. Once established, this coefficient allows for a reliable estimation of the tube diameter based on a measurement of the ratio *r*. The presented curve is the outcome of 87 tube diameter measurements. These diameters were grouped in increments of 5 nm, and the error bars represent standard deviations.

Alternately, the tube diameter can be estimated using fluorescence. The diameter of the tube is smaller than the optical resolution. However, for any given fluorescent lipid at a given surface density, the total fluorescence varies linearly with the membrane area. Hence, for a cylindrical tube, the intensity is proportional to the tube diameter. Once the proportionality coefficient is calibrated, a simple measurement of the tube intensity is sufficient to obtain a good and quick estimate of the tube diameter (Figure 3). This method is efficient when time is limited and a systematic measurement of the tube diameter by changing the length of the tube cannot be achieved.

Note that in the approach presented in this section, as well as in Section 3.3, there is no force measurement; hence, the actual BFP is not necessary and can be replaced with a micromanipulated bead without any erythrocyte. For instance, in Figure 2, no erythrocyte is present because it is not needed to determine the tube diameter (see Section 3.2 below).

### 3.3. Tube Diameter, Surface Tension and Bending Modulus

The micromanipulation of GUVs is convenient to accurately control the surface tension of the GUV membrane. This is very standard and has been known for decades. Again, the critical point is to avoid any adhesion between the micropipette and the GUV so that the tip of the membrane protrusion in the micropipette is a hemispherical cap with the same diameter as the micropipette, $d_{p}$. The shape of the GUV prior to tube formation is also a spherical cap with a diameter of $d_{v}$. Because of mechanical equilibrium, the surface tension along the membrane is uniform, and the Young–Laplace equation can be applied on both the membrane protrusion in the micropipette and the GUV outside of the micropipette.

(5) $$\left\{\begin{array}{c} P_{i}-P_{asp}=\frac{4\sigma }{d_{p}}\\ P_{i}-P_{sol}=\frac{4\sigma }{d_{v}} \end{array}\right.$$

where σ, $P_{i}$, $P_{asp}$ and $P_{sol}$ are, respectively, the membrane surface tension, the pressure inside the GUV, the pressure in the micropipette and the pressure in the solution. Subtracting both parts of Equation (5) directly provides σ as a function of the diameters and the aspiration in the micropipette, $\Delta P=P_{i}-P_{asp}$:

(6) $$\sigma =\frac{\Delta P}{4\left(\frac{1}{d_{p}}-\frac{1}{d_{G}}\right)}$$

Energetically, tubulation is costly because of the high curvature of the tube. This energy is provided by the mechanical displacement of the micropipettes. Upon separation of the micropipettes, when the bead is at short distance from the GUV surface, the GUV is slightly deformed. At larger distances, it is energetically more favorable to form a tube than to continue deforming the GUV [14]. This transition from GUV deformation to tubulation occurs when the deformation becomes larger than a couple of tube diameters. After tubulation, upon changing the surface tension by increasing or lowering the aspiration in the GUV micropipette, the tube diameter will vary. At higher aspiration, the tube diameter is smaller than at lower aspiration because the membrane is pulled towards the micropipette. When the diameter becomes too small, typically ~10 nm, the tube quickly breaks as fission is triggered by thermal fluctuations.

The relation between the tube diameter, the membrane surface tension and the bending modulus, Κ, can be obtained by considering the surface energy and curvature energy variations upon a small change in tube diameter, $\delta d_{t}$:

(7) $$\delta F=\pi L_{t}\left(-\frac{2Κ}{d_{t}^{2}}+\sigma \right)\delta d_{t}$$

At equilibrium, this variation is equal to zero; hence, the equilibrium diameter of the tube is:

(8) $$d_{t}=\sqrt{\frac{2Κ}{\sigma }}$$

This equation has often been used to measure bending moduli. In the example presented in Figure 2, the GUV diameter is 35 µm, the pipette diameter is 2.7 µm and the aspiration is 35 Pa. Hence, the surface tension is 26 µN/m. Since the tube diameter is ~82 nm, an estimate of the bending modulus value is 8.7 × 10^−20^ J, i.e., 20 *k_B_T*, which is in perfect agreement with previous measurements [36].

### 3.4. Force Required for Tubulation

In this section, we focus on the force aspect of tubulation, and the use of a BFP is absolutely required.

**Figure 4:**
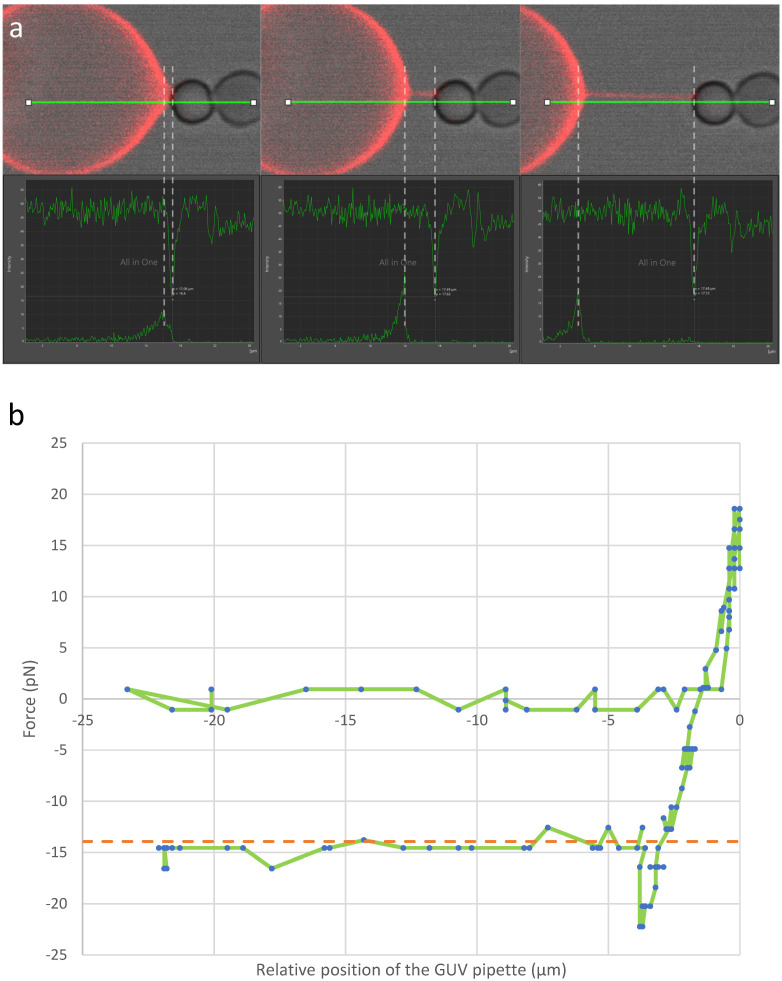
Force measurement. In (**a**), the three upper panels are screenshots extracted using Leica LasX software (version 4.5.0.25531) from the experiment featured in Video S4. The GUV comes into contact with the BFP and is subsequently moved away. The force is determined throughout this process by measuring the bead displacement and utilizing the predetermined stiffness of the BFP. Upon tube formation, a negative force is observed, indicating that the bead is being drawn towards the GUV. The three lower panels provide the intensities (a.u) observed along the green horizontal line (µm) in the bright-field channel (bead, top curve) and fluorescence channel (GUV, bottom curve) for the three screenshots in the upper panels. The red (resp. white) dashed lines between the screenshots and the intensity plots indicate the end of the tube on the GUV side (resp. the bead side) indicated by the maximum (resp. minimum) intensity at each timepoint. In the example illustrated in panel (**b**), the force is plotted against the relative position of the left pipette (green line). The leftmost panel in (**a**) corresponds to the point where the attractive force is maximal (approximately −20 pN), while the subsequent two panels correspond to the plateau around −15 pN at two distinct tube lengths. In this example, the surface tension of the GUV was 28.9 µN/m and the bending modulus 20.5 *k_B_T*. Hence, the tube force predicted by Equation (10) is 13.9 pN. This predicted tube force, indicated by the orange dashed line, is very close to the experimental value.

Tubulation is achieved by bringing the BFP bead in contact with the GUV and separating the micropipettes. As discussed in the previous section, during the course of micropipette separation, tubulation from the GUV is a two stage-process (Figure 4a and Video S4). First, the GUV is deformed. This first regime is an elastic regime, and the force increases linearly with the deformation at the level of the bead-GUV contact point. The elastic constant driving this deformation is proportional to the membrane surface tension, σ. The proportionality coefficient has been predicted to be $2\pi /\left(ln\left(2\right)-\gamma \right)\sim 54.2$, where γ is the Euler constant [14]. Hence, theoretically, the precise measurement of deformation enables accurate determination of the membrane surface tension within this specific regime. Such a measurement is delicate (see end of this section). Hence, it is often easier to use the aspiration in the micropipette and Equation (6) to have a sufficiently accurate value of σ.

When the force and deformation reach a certain threshold, the formation of a tube becomes energetically more favorable. At this maximum elastic deformation, the force peaks. The start of tubulation is associated with a sudden 11–12% reduction in the force. When the micropipettes’ separation is subsequently increased, the tube is elongated but the force remains approximately constant. The value of the force, $f_{t}$, can be obtained by considering the free energy variation upon a change in the tube length, $\delta L_{t}$:

(9) $$\delta F=\left(\pi L_{t}d_{t}\left(\frac{2Κ}{d_{t}^{2}}+\sigma \right)-f_{t}\right)\delta L_{t}$$

At mechanical equilibrium, this variation is equal to zero. Hence,

(10) $$f_{t}=\pi d_{t}\left(\frac{2Κ}{d_{t}^{2}}+\sigma \right)=2\pi \sqrt{2Κ\sigma }$$

This two-stage tubulation process has been observed using optical tweezers [9]. The BFP is also well-suited for quantitatively monitoring tubulation and determining tube diameter, bending moduli and membrane surface tension, as demonstrated in Figure 4. Analyzing the curve in Figure 4b reveals a 25–30% decrease in the GUV pulling force on the BFP—from the maximum value at the transition between the two stages, approximately 20–25 pN, to the plateau force during tubulation, around 15 pN. This observed relative decrease aligns with findings from optical tweezers but surpasses the predicted decrease, which may stem from experimental artifacts, such as imperfect equilibrium due to kinetics, or theoretical approximations, such as assuming a perfectly flat membrane. In the experiment presented in Figure 4b, the surface tension was 28.9 µN/m and the bending modulus 20.5 *k_B_T*. Hence, the plateau force predicted during tubulation was 13.9 pN, which is consistent with the observed force of ~15 pN, confirming the validity of Equation (10).

Regarding the slope in the force/extension curve in the initial phase, accurate determination proves challenging due to the difficulty in precisely measuring the actual extension of the GUV, as defined in the model [14]. However, approximating the force/extension slope by subtracting the erythrocyte stiffness from the graph in Figure 4b yields a difference of approximately 100 µN/m. This difference suggests a surface tension of around 2 µN/m, which is roughly ten times underestimated. This outcome does not invalidate the theory but underscores the challenges in accurately measuring the actual GUV deformation.

## 4. Discussion

In this article, we demonstrated that tubulation from GUVs, commonly accomplished through a combination of micromanipulation and optical tweezers, can be achieved using exclusively micromanipulations. Both approaches exhibit identical capabilities and potential. The benefits of employing the BFP are twofold. First, constructing the micromanipulation system is more cost-effective. Second, the spring constant can be easily determined through straightforward optical measurements (pipette diameter, erythrocyte diameter, erythrocyte/bead contact diameter) and the accurate determination of zero pressure and reservoir height, eliminating the need for specific calibration. Hence, once the probe is assembled, measurements can commence immediately.

In addition to precisely determining the bending and stretching modulus of the membrane, real-time force monitoring can offer direct insights into the actions of certain proteins by correlating their binding with alterations in the GUV or tube mechanical properties or composition.

It is noteworthy that the utilization of a BFP for tubulation is not confined to GUVs. Substituting the GUV with a live cell is feasible, and measuring forces during cell tubulation can provide information about the local mechanical properties of the cell. Video S5 illustrates an example of a tube originating from a K562 cell.

Furthermore, it is viable to affix a rigid substrate to the BFP bead and generate tubes between this rigid substrate and the bead. For instance, a prior study explored tubulation between a spermatozoon bound to the bead and an oocyte [26]. This approach enabled mapping of the mechanical behavior of the oocyte and determination of how the local mechanical properties of the oocyte impact interactions with the spermatozoon. Conducting such a study with optical tweezers could have been more challenging, as the spermatozoon might not withstand the laser beam.

Often, when cells are used, a sudden decrease in force between the deformation and tubulation stages is not observed [24,26]. This difference between model membranes and live cells probably originates from the presence of the cytoskeleton and/or the local viscosity of both the membrane and the cytosol.

## Data Availability

The data are contained within the article and Supplementary Material.

## Supplementary Materials

The following are available online at https://www.mdpi.com/article/10.3390/membranes13120910/s1, Video S1: Determination of $h_{0}$ corresponding to the “zero” aspiration. A micrometric dirt particle is aspirated inside the micropipette and initially positioned on the left-hand side of the micropipette. The height of the reservoir is gradually adjusted above $h_{0}$, causing the particle to move towards the tip of the micropipette. Subsequently, the height is lowered below $h_{0}$, causing the particle to move back to the left side of the micropipette. This blowing/suction cycle is repeated three times in the video until the reservoir height aligns precisely with $h_{0}$, a few seconds before the video concludes, as evidenced by the immobilization of the particle. This process is highly sensitive, with height variations in the video remaining within a 200 µm range. The entire video unfolds in real time, providing an accurate representation of the dynamics involved in determining $h_{0}$. Video S2: BFP formation. The left micropipette holds a streptavidin-coated bead, which is then brought into contact with a biotinylated erythrocyte held by the right micropipette. After a few seconds, the bead is released, leading to the assembly of the BFP. Maintaining a geometry as close as possible to axial symmetry is crucial. The entire process is presented in real time and is associated with the content depicted in Figure 1. Video S3: Tube diameter measurement. A tube is generated between the giant unilamellar vesicle (GUV) in the left pipette and the streptavidin bead in the right pipette. The bead is manipulated towards the GUV to decrease the tube length, followed by movement away from, and then, towards the GUV once more. Throughout this process, changes in the length of the GUV extension inside the left pipette are attributed to the tube membrane and volume released (approach) or added (separation). The video is accelerated approximately 20 times. Video S4: Force measurement. When a GUV (left) initially placed in contact with the BFP is moved away from the BFP, a tube forms and exerts a force that extends the BFP. The extension is proportional to the force through the nanospring stiffness obtained from Equation (1). Video S5: Tubulation between the BFP and a living cell. The BFP bead was coated with a biotinylated penetratin peptide, which inserts into the cell membrane. Upon contact between the BFP bead and a K562 cell, a tube occasionally formed during the retraction of the BFP. The existence of the tube was demonstrated and visualized by the movement of the cell, mirroring the displacement of the BFP micropipette, even though no apparent physical contact was observed. Towards the end of the video, the tube was stretched excessively, leading to its rupture. Following the breakage of the tube, the BFP probe spontaneously returned to its resting position with axial symmetry.
